# Monocytes and B cells support active replication of Chandipura virus

**DOI:** 10.1186/s12879-016-1794-6

**Published:** 2016-09-14

**Authors:** Soumen Roy, Daya Pavitrakar, Rashmi Gunjikar, Vijay M. Ayachit, Vijay P. Bondre, Gajanan N. Sapkal

**Affiliations:** National Institute of Virology, Sus Road, Pashan, Pune, 411021 India

**Keywords:** Chandipura virus, PBMC, Monocytes, B cells, Cytokine, Chemokine

## Abstract

**Background:**

Interaction between immune system and Chandipura virus (CHPV) during different stages of its life cycle remain poorly understood. The exact route of virus entry into the blood and CNS invasion has not been clearly defined. The present study was undertaken to assess the population in PBMC that supports the growth of virus and to detect active virus replication in PBMC as well as its subsets.

**Methods:**

PBMC subsets viz.: CD3^+^, CD14^+^, CD19^+^, CD56^+^cells were separated and infected with CHPV. The infected cells were then assessed for transcription (N gene primer) and replication (NP gene primer) of CHPV by PCR. The supernatant collected from infected cells were titrated in Baby Hamster Kidney (BHK) cells to assess virus release. The cytokine and chemokine expression was quantified by flow cytometry.

**Results:**

Amplification of N and NP gene was detected in CD14^+^ (monocyte) and CD19^+^ (B cell), significant increase in virus titre was also observed in these subsets. It was observed that, although the levels of IL-6 and IL-10 were elevated in CD14^+^ cells as compared to CD19^+^cells, the differences were not significant. However the levels of TNFα and IL-8 were significantly elevated in CD14^+^ cells than in CD19^+^cells. The levels of chemokine (CXCL9, CCL5, CCL2, CXCL10) were significantly elevated in CHPV infected PBMC as compared to uninfected cells. CCL2 and CXCL9 were significantly increased in CHPV infected CD14^+^cells as compared to CD19^+^ cells.

**Conclusion:**

CD14^+^and CD19^+^cells support active replication of CHPV. High viral load was detected in CD14^+^ cells infected with CHPV hence it might be the primary target cells for active replication of CHPV. An elevated levels of cytokines and chemokines observed in CD14^+^ cells may help in predicting the pathogenecity of CHPV and possible entry into the central nervous system.

## Background

Chandipura virus (CHPV) belongs to family *Rhabdoviridae*, genus *Vesiculovirus.* The family consists of several important human, animal and plant pathogens like rabies virus, vesicular stomatitis virus (VSV), potato yellow dwarf virus and Isfahan virus, etc. It is bullet shaped and consists of a linear, single stranded negative sense RNA molecule of approximately 11,120 base pairs [1, 2]. CHPV was first isolated from the serum of a patient with febrile illness in Chandipura village near Nagpur, Maharashtra in India during an investigation of Chikungunya outbreak [3, 4]. CHPV was incriminated as the etiological agent of large-scale encephalitis outbreaks in children with high case fatality rate in various districts of Andhra Pradesh, Gujarat and Maharashtra indicating its disease causing potential [4, 5].

Viruses belonging to the *Vesiculovirus* genera share similar genomic and structural organization [6, 7]. The molecular regulations of their replication and transcription events are also comparable. The five transcriptional units are transcribed by a single promoter at the 3′end and code for the nucleocapsid protein (N), phosphoprotein (P), matrix protein (M), glycoprotein (G) and polymerase large protein (L). The RNA genome is tightly encapsidated by the viral N protein to form helical ribonucleoprotein complex (RNP). This encapsidated genome serves as template for both replication and transcription. One of the molecules that have been implicated in the trasnscription-replication switch is the viral N protein. The intracellular concentration of N protein modulates the transition of polymerase action from transcription to replication by encapsidating the nascent leader RNA and thereby suppressing the intergenic transcription termination signals. In VSV, it was suggested that N-P complex formation could be the key mechanism for this switch. Infected cell extract immunodepleted of N-P complex with an antibody against P protein was unable to support replication in an in vitro assay. All these reports strongly suggest a possible auxiliary function of P protein in viral replication [8–11]. Recently it has been shown that P protein undergoes considerable structural changes upon Leader (Le) RNA binding thus forming functional replicase complex L-N-P-Le-RNA [12].

Sandfly (*Phlebotomus papatasi*) acts as a vector for CHPV transmission which has been confirmed in murine model experimentally [13]. The studies in the murine model reveal that blood cells are attractive targets for CHPV as they are located mainly in the circulation and causes acute inflammation followed by dissemination into the central nervous system [14, 15]. CHPV was isolated from patient’s leukocytes suggest that blood leukocytes could be infected and involved in viral production [4]. The exact route of entry to the susceptible cells in the central nervous system (CNS) and the events taking place during the acute blood phase of CHPV infections has not been clearly understood. Interactions between immune system during CHPV life cycle remains poorly defined. Although the role of cytokine and chemokine is unclear, increased levels of certain cytokines has been observed in CHPV related encephalopathy in children [16]. However the interaction of peripheral blood mononuclear cells (PBMC) and its subsets with CHPV is unclear. In this study we tried to understand the susceptibility of PBMC and its subtypes to CHPV infection. We have also assessed cytokine and chemokine levels from the infected cell population which could give us the insight of CHPV pathogenesis.

## Methods

### Cell line and virus

Baby hamster kidney (BHK) cell line was maintained in Minimal essential medium (MEM, Sigma) with 10 % fetal calf serum (FCS; Gibco). Chandipura virus (CHPV) used in this study was (CIN6514) isolated in 1965 [3]. The viral stock was prepared in BHK cells and stored at −80 °C for further use. The inactivated CHPV was prepared by UV treatment. Briefly tissue cultured grown CHPV virus was exposed to UV for 40 min. The virus infectivity was confirmed with three blind passages in BHK cells. Titration of the virus stock was carried in BHK cells by plaque assay. The virus titre was determined as plaque forming units per milliliter (pfu/ml) or Tissue culture infective dose _50_(TCID_50_/ml) to calculate multiplicity of infection (MOI) in infection experiments [17].

### PBMC isolation and cell separation

Buffy coat packs from adult healthy anonymous donors (*N* = 9) were obtained from a hospital blood bank, (Pune, India). Since the informed consent was obtained from voluntary blood donors at the time of blood donation, the institutional human ethical committee, (National Institute of Virology, Institutional human ethics committee) confirmed that no further approval is required for using anonymized leftover specimens/components from blood donations of the local blood bank for scientific purposes. The samples were tested for presence of anti-CHPV antibodies before use. PBMCs were isolated by Ficoll Hypaque (Sigma, USA) gradient centrifugation and resuspended in RPMI 1640 medium with 10 % FCS. Magnetic Activated Cells Sorting (MACS) [Miltenyi Biotec, Inc. Auburn, CA] was used for separation of CD3^+^ (T lymphocytes), CD14^+^ (Monocytes) and CD19^+^ (B lymphocytes), CD56^+^ (NK cells) cells from whole PBMC as per manufacturer’s guidelines [18].

### Determination of cell purity

Various cell population *viz.* CD3^+^, CD14^+^, CD19^+^, CD56^+^ were sorted using MACS and re-suspended in PBS. Specific phycoerythrin (PE) conjugated monoclonal antibodies were added to each subset and incubated at 4 °C for 30 min. The cells were washed and fixed with 1 % paraformaldehyde in PBS and were analyzed using Cell Quest Pro software in FACS-calibur (BD Bioscience, USA). The percentage purity of isolated cells was found to be in the range 95 to >96 %.

### In vitro infection and quantification

The PBMC and CD3^+^, CD14^+^, CD19^+^, CD56^+^ cells were infected with CHPV in complete medium (RPMI + 10 % FCS). Briefly, 0.2 x 10^6^ to 1x10^6^ PBMC and its subsets were infected with viral suspension for 1 h at 37 °C, in 5 % CO_2_. The cells were washed thrice with PBS, and replenished with complete medium. Uninfected cell controls as well as cells infected with UV inactivated virus were also treated in similar way. Cell culture supernatant and cell lysates were collected at defined time points to determine virus growth kinetics and cytokine levels. The virus titre in the culture supernatant was estimated. Briefly, serial tenfold virus dilutions prepared in MEM with 2 % FBS was added in triplicate to the BHK cells. The plate was incubated at 37^°^C with 5 % CO2, and observed daily for cytopathic effect. After 72 h, the assay was terminated and stained with 1 % amido black. The virus titers were expressed as TCID_50_/ml) [17].

### Detection of CHPV transcriptive and replicative intermediate

CHPV infected PBMC, CD3^+^, CD14^+^, CD19^+^, CD56^+^ and mock controls were harvested at different time intervals post infection. CD19^+^cells were treated with 0.01 % of sodium azide and infected with CHPV to analyze for replicative intermediate for ruling out the possibility of the uptake of CHPV was not due to capping of immunoglobulin. RNA was extracted from cell lysate by using TRIzol® RNA extraction method (Life Technologies) as per manufactures instruction. One step Reverse transcriptase polymerase chain reaction (RT-PCR) was performed using kit containing 2X PCR mix and SuperScript® III Reverse transcriptase/Platinum^®^*Taq* DNA polymerase (Invitrogen CA, USA). One microgram of total RNA was subjected to one step RT-PCR with different primer pairs corresponding to the following:

i) upstream (5’-CGG AGA GAA ATG TTG TTG TGT G-3’) and downstream (5’-GCA AAG AGT TTC CTG GCG TA-3’) primers specific for a 150 bps amplicon of the N gene, used for transcription assessment. For replication assessment ii) upstream (5’-TGG AAA GGG TAG GAG ATA TTC GA-3’) and downstream (5’-GAG AGT GTC CTG AAG CTT TGG-3’) primers specific for a 150 bps amplicon of the N-P gene junction specific primers were used [12]. The RT-PCR products were resolved on 1.8 % agarose gel and visualized by staining with ethidium bromide.

### Determination of pro-inflammatory cytokine and chemokine levels

Pro-inflammatory cytokine and chemokine concentration in the CHPV infected and mock infected culture supernatant and un-infected cell supernatant control were determined using a cytometric bead array (CBA) kit (BD, Biosciences, USA) on a FACS Calibur with appropriate controls. The acquired data was analyzed using FCAP array 2.0 software and each sample was tested in triplicate.

### Statistical analysis

Wilcoxon signed-rank test was used to determine modulations in chemokine levels. Paired test was applied to compare cytokine and chemokine levels among CHPV infected PBMC, CD14^+^cell, CD19^+^cell supernatant and respective uninfected cell control (CC). Differences with *p ≤* 0.05 were considered statistically significant.

## Results

### PBMC support active replication of CHPV RNA

During CHPV life cycle, N gene is the earliest gene transcribed after viral entry. Whereas during virus replication phase tripartite complex (N-P-L) is synthesized and could be detected by N-P junction primers. Detection of N-P gene thus assures that signature transcription termination sequence (characteristic of all Rhabdoviruses) is ignored and viral replication is initiated. Therefore, the detection of N gene and NP gene could be used as an index to determine transcription and replication respectively.

**Fig. 1:**
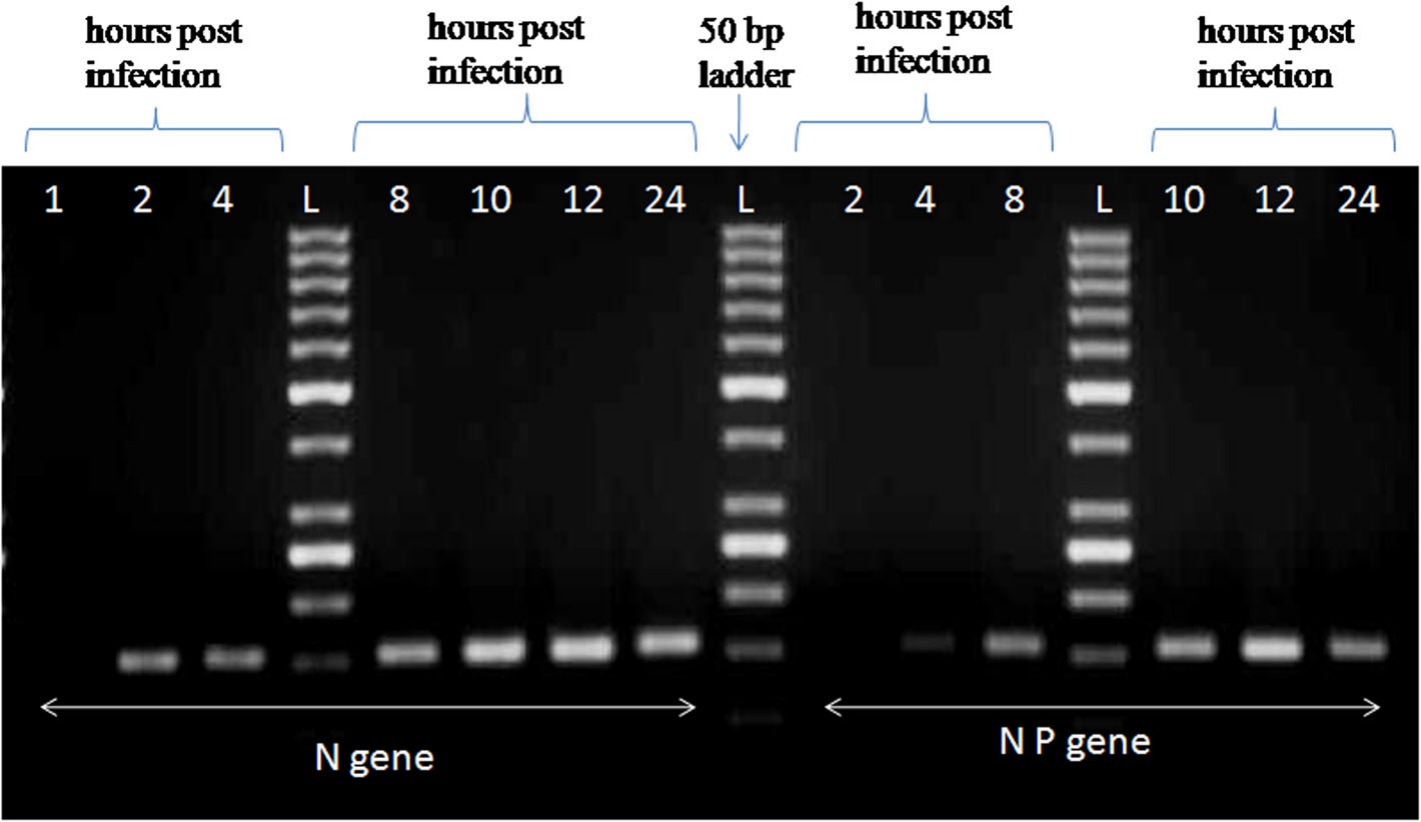
Detection of transcriptive and replicative form of CHPV in human PBMC. The PBMC’s were isolated from the whole blood cells of healthy donors and infected with MOI = 5 CHPV in complete medium (RPMI + 10 % FCS). Cells were harvested at different hours time interval (1, 2, 3 , 8, 12 and 24 h) and total RNA was extracted by Trizol method. The transcriptive form (N gene) and replicative form (NP gene) was detected by RT-PCR. The assay was performed in triplicates

In order to investigate whether PBMC supports the active CHPV replication, PBMC from the nine healthy donors were infected with CHPV at different MOI (MOI = 1, 2 and 5). Cell pellets were harvested at various time intervals and analyzed for replicative and transcriptive intermediates of CHPV using N gene (replicative) and NP gene (transcriptive) primers by one step RT-PCR. There was no amplification of N and NP gene was observed when infected with MOI = 1 and MOI = 2. Whereas 150 bp amplicon was detected for N gene (transcription) at 2 h post infection (pi) with MOI = 5 of CHPV. While NP gene (replication) was detected at 4 h pi onwards when infected with MOI = 5 CHPV (Fig. 1).

**Fig. 2:**
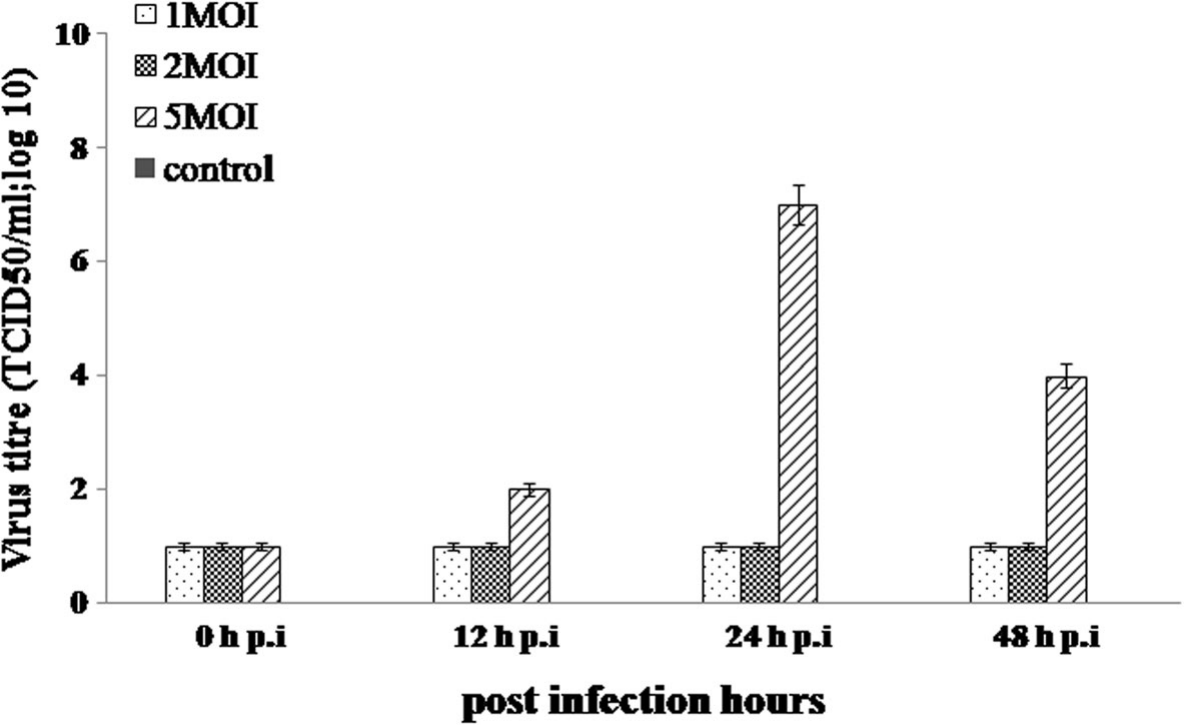
Replication kinetics of CHPV in human PBMC. The PBMC’s from healthy donors were infected with CHPV at MOI = 1, MOI = 2 and MOI = 5 along with the controls. The culture supernatant was collected at different time interval (0, 12, 24 and 48 h) post infection. The release of progeny virus was quantitated by calculating TCID_50_ titre in BHK cells

To determine the extent of replication in PBMC, the culture supernatants collected at various time intervals were analyzed for CHPV replication kinetics. There was no increase in the virus titer with MOI = 1 and 2 whereas with MOI = 5 there was a gradual increase in virus titre at 12 h pi, it increased up to 2 log TCID_50_ and reached its peak up to 7 log TCID_50_ after 24 h pi. At 48 h pi, the virus titre declined to 4 log TCID_50_ (Fig. 2).

### CHPV target cell(s) support CHPV replication

**Fig. 3:**
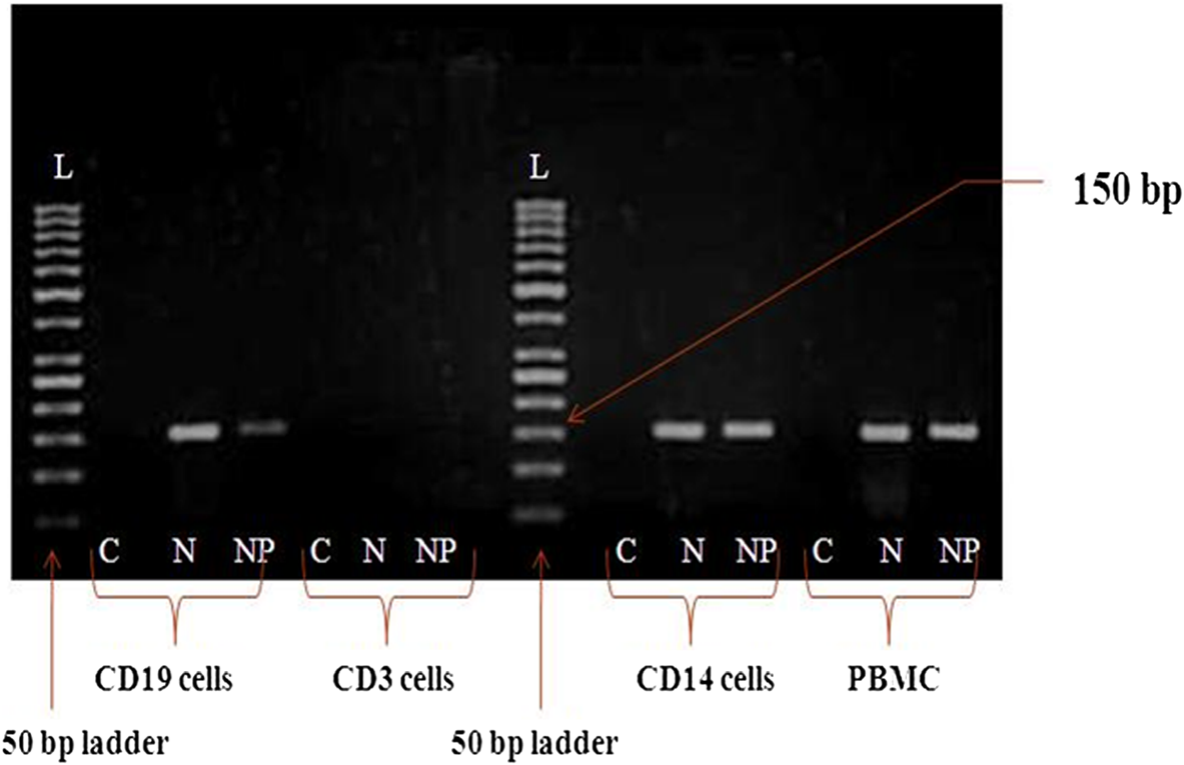
Detection of transcriptive and replicative form of CHPV in human PBMC subsets. The PBMC was isolated from the whole blood cells healthy donors. CD3^+^,CD14^+^,CD19^+^ cells were separated from PBMCs by MACS separation method and were infected with CHPV at MOI = 5 in complete medium (RPMI + 10 % FCS). Cells were harvested at 24 h pi and total RNA was separated by Trizol method. The transcriptive form (N gene) and replicative form (NP gene) was detected by RT-PCR. The assay was performed in triplicates. Cell control is represented as C in the figure

To determine which subsets of PBMC supports the CHPV replication, CD3^+^, CD14^+^, CD19^+^and CD56^+^cells were separated from PBMC and each were infected with MOI = 5 of CHPV. The N gene and NP gene was not amplified in CD3^+^cells and CD56^+^cells. Analysis of CD14^+^ and CD19^+^ cells revealed N gene and NP gene amplification corresponding to band size of 150 bp indicating that CD14^+^and CD19^+^ cells support CHPV replication. There was no amplification of N gene and NP gene observed in cell subsets infected with UV inactivated CHPV. Overall these results indicate that CD3^+^cells and CD56^+^cells did not support active replication while CD14^+^ cells as well as CD19^+^support CHPV growth (Fig. 3).

### Comparative analysis of CHPV replication in CD14^+^ and CD19^+^ cells

**Fig. 4:**
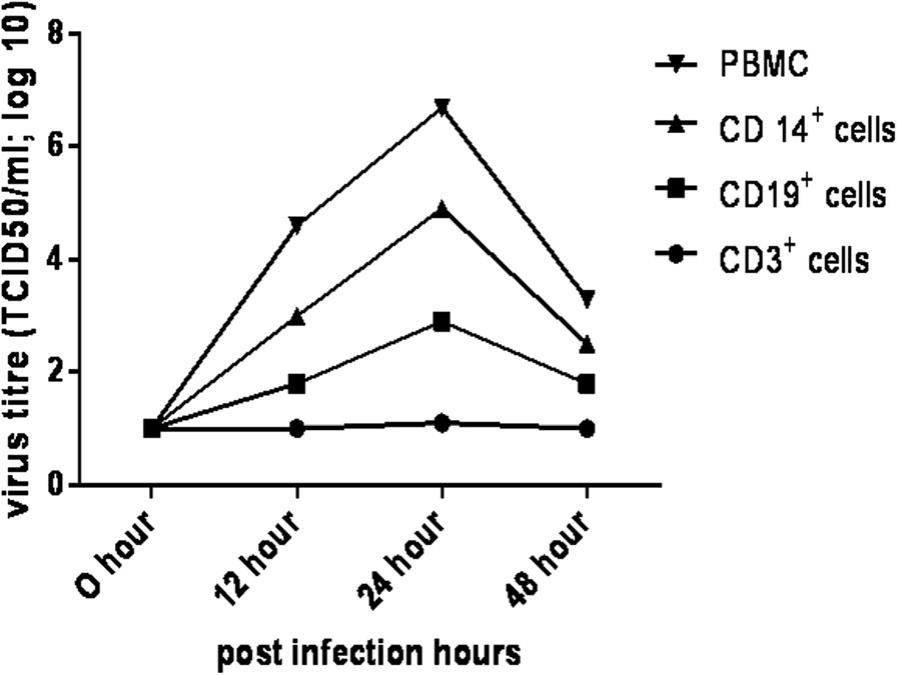
Replication kinetics of CHPV in human PBMC subsets () PBMC, () CD14^+^cells, () CD19^+^cells and () CD3^+^cells infected with MOI = 5 of CHPV. The culture supernatant was collected at different time interval. The release of progeny virus was quantitated by calculating TCID_50_ titre in BHK cells. The assay was performed in triplicates

Since the replicative intermediates were detected in both CD14^+^ (monocyte) cells and CD19^+^ (B cells) comparative viral replication kinetics were assessed for infectious virion release from these subsets. The release of virus from CD3^+^cells, CD56^+^cells was lower and remained at the basal level throughout the study indicated that these cell subsets did not support active replication of CHPV. Whereas there was a gradual increase in virus titre that peaked at 24 h pi up to 5 log TCID_50_ in CD14^+^ cells which was found to be higher than CD19^+^ cells with 3 log TCID_50_. At 48 h pi the virus replication declined (Fig. 4).

### Pro inflammatory cytokine and chemokine analysis

**Fig. 5:**
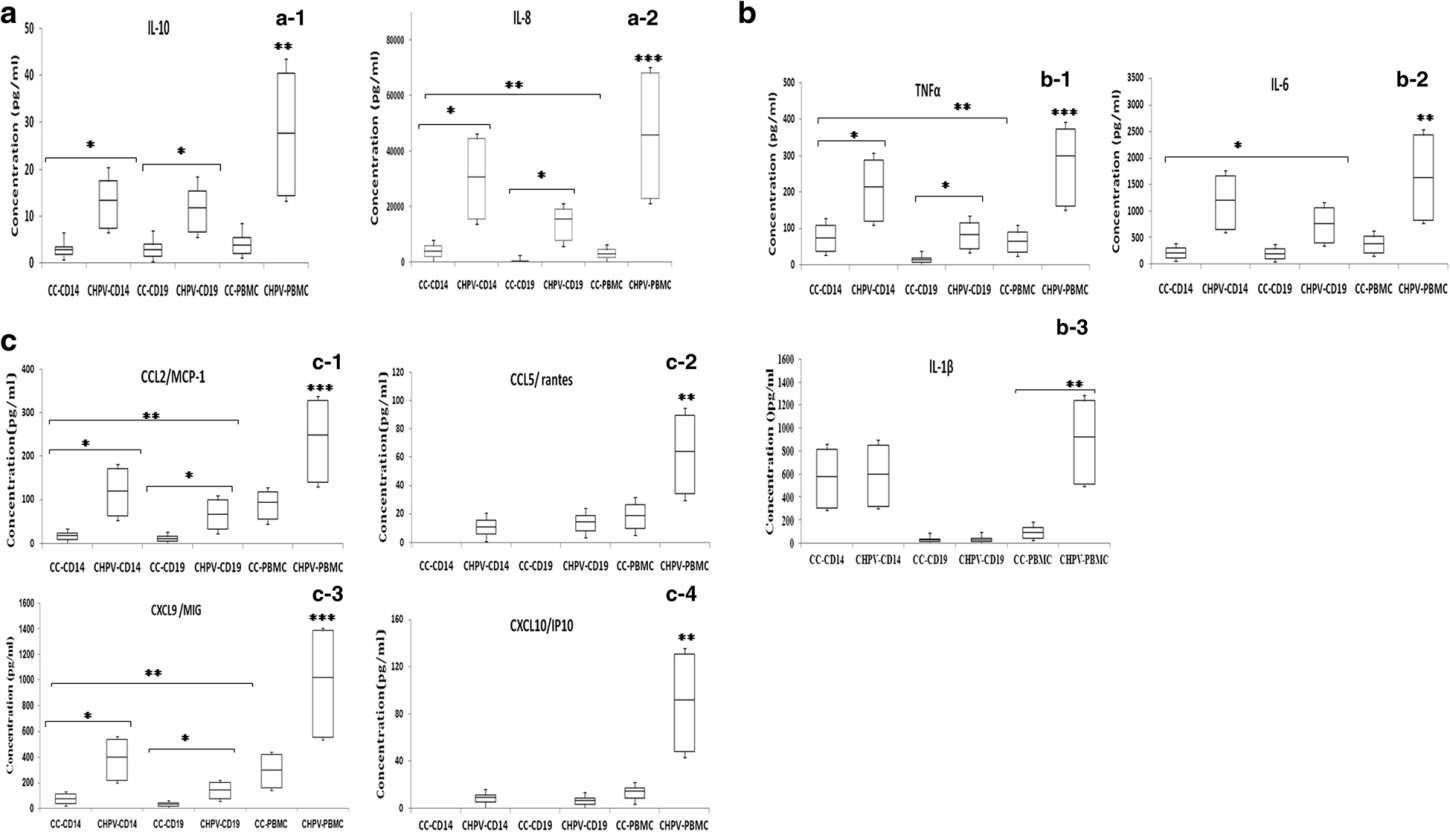
Cytokine and Chemokine levels in CHPV infected human PBMC subsets. **a** Pro-inflammatory cytokine levels (IL-10, IL-8) in CHPV infected PBMC, CD14^+^cell, CD19^+^cell supernatant. **b** Pro-inflammatory cytokine levels (TNFα, IL-6, IL-1β) in CHPV infected PBMC, CD14^+^cell, CD19^+^cell supernatant and respective un infected cell control (CC) at 24 h post infection (pi). **c** Chemokine levels (CCL2, CCL5, CXCL9, CXCL10) in CHPV infected PBMC, CD14^+^cell, CD19^+^cell supernatant and respective un infected cell control (CC) at 24 h post infection. The box plot represents minimum, first quartile, median, third quartile and maximum. Total of 9 donors were used in the experiment. Statistical analysis was performed by Wilcoxon signed rank test. Paired test was applied to compare cytokine and chemokine levels among CHPV infected PBMC, CD14^+^cell, CD19^+^cell supernatant and respective uninfected cell control (CC). *p* value < 0.05 (*) significant, (**) 0.005 moderately significant and (***) 0.0005 highly significant among groups

Levels of pro-inflammatory cytokine (IL-1β, IL-6, IL-8, IL-10, and TNFα) were found to be elevated and significantly higher among infected PBMC as compared to their respective controls. Levels of IL-6, IL-10,IL-8 and TNFα were found to be significantly increased in CD19^+^cell (B cells) as compared to its controls (*p* < 0.005). There was no significant difference in IL-1β levels from CHPV infected B cells and its control. IL-6, IL-8, IL-10 and TNFα levels were found to be significantly increased in CD14^+^cells (monocytes) as compared to its controls (*p* < 0.005) (Fig. 5a, b). The difference in IL-1β levels in CHPV infected CD14^+^cells and its controls were not significant. Although the levels of IL-6 and IL-10 were elevated in CD14^+^ cells as compared to CD19^+^cells, the differences were not significant. However the levels of TNFα and IL-8 were significantly elevated in CD14^+^ cells than in CD19^+^cells (*p* < 0.005). The levels of chemokine (CXCL9, CCL5, CCL2, CXCL10) were significantly elevated in CHPV infected PBMC as compared to uninfected cells. CCL2 and CXCL9 were significantly increased in CHPV infected CD14^+^cells as compared to CD19^+^ cells (Fig. 5c).

## Discussion

These studies were initiated to determine the contribution of PBMC subsets to CHPV amplification and cytokine secretion in triggering the innate immune response. There are no animal models that mimic the clinical syndrome of severe encephalitis in humans and therefore observational studies of patient cohorts are the main source of understanding immunopathogenesis of CHPV infection [5, 16]. Limited studies in mice revealed that CHPV induces viremia followed by dissemination of the virus to secondary infection sites and reach the CNS. During peripheral circulation, blood leukocytes such as monocytes, dendritic cells and natural killer (NK) cells are the main components of innate immunity and they have been implicated in the immunopathogenesis of many viral diseases. These cells are attractive virus targets because they are located mainly in the circulation and peripheral tissues moreover they can assist virus dissemination [14, 15].

Our result demonstrates that CHPV replicate in monocytes as well as in B cells and trigger cytokine response. In vitro model of unfractionated PBMC infected with CHPV indicated lodging of virus. Although the intracellular positive staining for CHPV was observed, very low numbers of cells were infected even at high viral load (MOI = 10) and hence no significant conclusions could be drawn (flow cytometry, data not shown). In order to assess the replication in infected cells, the virus release and the replicative intermediates were analyzed. Consistent to earlier report by A. Roy et al. [12], the transcriptive intermediate (N gene) was detected after 2 h pi and replicative intermediate (NP gene) were detected after 4 h pi in PBMC infected with CHPV. It has been proposed that during CHPV transcription, progressive termination at each gene junction produce the positive sense leader RNA and 5′mRNA corresponding to 5 viral genes that are N, P, M, G and L [2, 7]. The detection of N gene amplification indicated the positive strand RNA genome i.e., transcription in the infected cells. During replication, the gene junction termination signals are ignored to produce positive strand polycistronic RNA complementary to the whole genome which then serves as the template for the synthesis of progeny genome RNA [2, 7, 12]. Data represented here further revealed the NP gene amplification which is the replication template present only during virus replication, was found in infected cells. To determine the extent of replication in PBMC, infectious virus titres were determined using supernatants collected at various time points post infection. It was observed that the amount of virus detected in infected cells increased over time. The titre peaked at 24 h pi which confirms that PBMC support CHPV replication.

We further extended our studies to pinpoint the target cell(s) of CHPV infection in PBMC. PBMC consist of roughly 70 % T cells, 10 % B cells, 10 % Monocytes and 1 % NK cells [19]. The CD3^+^ (T cells), CD14^+^ (Monocytes), CD19^+^ (B cells), CD56^+^ (NKcells) were infected with MOI = 5 of CHPV. Monocyte and B cell support active replication of CHPV. The replication of CHPV observed in monocytes or B cells was not due to the contamination of either cell as subsets used for studies contained high percentage of monocyte/B cell. Infected cells coexpressed the cell markers (CD14/CD19) and viral antigens. One of our key findings is that primary monocyte without stimulation or differentiation was permissive for CHPV replication. The titres of virus released from the infected monocytes (5 log TCID_50_) were found significantly higher than B cells (3 log TCID_50_). It was reported that B cell line (Raji) adapted dengue virus could replicate in B lymphocytes in in vitro infection model [20, 21]. The CHPV used in the current study was not grown /adapted in any B cell and monocyte cell line or PBMC. Cells treated with sodium azide exhibited the presence of both N and NP gene amplification. Thus the efficiency of infection probably reflects viral entry receptors expressed on these cells that requires further studies. Studies on other members of *Rhabdoviridae* family such as VSV and rabies virus demonstrated that monocytes support replication of these viruses. It was also reported that rabies virus infects murine macrophages (IC-21 and bone marrow derived macrophages) as well as human macrophage cell (U937 and THP-1) in-vitro [19, 22–24]. Several studies on arboviral model suggested that blood leukocyte such as monocytes and B cells support viral growth. Chikungunya virus actively replicates in monocytes whereas in dengue virus, monocytes are the primary cells among PBMC, while B lymphocytes also harbours and supports dengue virus infection [17, 20, 21, 25]. Overall we observed that monocytes and B cells are permissible for CHPV growth. The experimental conditions used thus reflect more closely the environment where these subsets may encounter in vivo virus infection. The modulation of the cytokine and chemokine responses during the course of CHPV has not been fully elucidated. It has been shown that during early infection (within 24 h) there is pro- and anti-inflammatory response to CHPV infection in vitro; however, the dynamics of cytokine and chemokine response during the course of CHPV replication in human is unknown. We further investigated the ability of purified monocyte/ B cells to trigger the cytokine and chemokine response after CHPV infection. Elevated levels of IL-1β, IL-6, IL-8, IL-10 and TNFα were observed in CHPV infected PBMC as compared to their mock controls. IL-6, IL-10, TNFα levels were significantly higher in monocytes as compared to B cells.

The elevated levels of IL-6, IL-10, and TNFα have been suggested to play a role in the pathogenicity of CHPV in mice model [14]. In addition similar observations were made in patients with CHPV encephalitis [14, 16, 26]. These results corroborate our findings that CHPV replication favors the induction of pro-inflammatory response.

CHPV infection of PBMC, Monocytes and B cells strongly stimulated the chemokines (CCL2/MCP-1, CCL5/rantes, CXCL9, CXCL10/IP10) during the course of infection. Elevated levels of CCL2, CCL5, CXCL9, and CXCL10 were observed in infected monocytes and B cells as compared to their controls. However, the secreted levels of CCL2 and CXCL9 were significantly higher in monocytes as compared to B cell. Although the role of chemokines in CHPV infection is unclear, the increased levels of CCL2 during CHPV infection in mice model might play a role in increasing the permeability of blood brain barrier (BBB) to allow virus into CNS [14, 15]. Increased level of CCL2 might also explain the accumulation of lymphocytes and macrophages in the spleen, lymph nodes and other tissue [27].

Various studies found upregulation of CCL2, CCL3, CCL5, CXCL9, and CXCL10 in CNS after rabies virus infection suggested being responsible for the increased infiltration of inflammatory cells and BBB permeability [28, 29]. Moreover it was also reported that CCL2 is responsible for increased permeability and transmigration of HIV infected human leukocytes across BBB in in vitro model [30]. Here the up regulations of CCL2 and CXCL9 in CHPV infected monocytes were observed. Changes in cytokine and chemokine levels during the course of CHPV infection could have a major impact on the immune system; influencing pathogenesis by enhancing or suppressing CHPV replication as well as favouring the formation of infected cell reservoirs in CNS needs to be determined.

## Conclusion

From this study we concluded that monocytes and B cells support active replication of CHPV, high viral load was detected in monocytes infected with CHPV. The elevated levels of IL-10, IL-6, IL-8, TNFα (cytokines) and CCL2 and CXCL9 (chemokines) in monocytes may help in predicting the pathogenicity of CHPV causing encephalitis and possible entry into the central nervous system. This was the first reported data of the cytokine and chemokine profile of CHPV.
